# Developmental expression profile of the *yy2 *gene in mice

**DOI:** 10.1186/1471-213X-9-45

**Published:** 2009-07-28

**Authors:** David Drews, Martin Klar, Christof Dame, Anja U Bräuer

**Affiliations:** 1 Department of Neonatology, Charité – Universitätsmedizin Berlin, Augustenburger Platz 1, 13353 Berlin, Germany; 2 Institute of Cell Biology and Neurobiology, Charité – Universitätsmedizin Berlin, Philippstraße 12, 10115 Berlin, Germany; 3 Department of Biology, Chemistry and Pharmacy, Freie Universität Berlin, Takustraße 3, 14195 Berlin, Germany

## Abstract

**Background:**

The transcription factor Yin Yang 2 (YY2) shares a structural and functional highly homologue DNA-binding domain with the ubiquitously expressed YY1 protein, which has been implicated in regulating fundamental biological processes. However, the biological relevance of YY2 has not been identified yet.

**Results:**

Towards the understanding of YY2 biology, we analyzed in detail the expression pattern of *yy*2 in various organs during embryonic and postnatal mouse development till adulthood. Thereby, a constant *yy2 *level was detected in heart and lung tissue, whereas in different brain regions *yy2 *expression was dynamically regulated. Interestingly, in any analyzed tissue neither the homologue *yy1 *nor the *mbtps2 *gene showed changes in mRNA expression levels like *yy2*, although the intronless *yy2 *gene is located within the *mbtps2 *locus.

Furthermore, we detected *yy1*, *yy2*, and *mbtps2 *mRNA in primary mouse neurons, microglia cells, and astrocytes. In comparison to *yy2 *and *mbtps2*, *yy1 *revealed the highest expression level in all cell types. Again, only *yy2 *showed significantly altered gene expression levels among the cell types. Higher *yy2 *expression levels were detected in microglia cells and astrocytes than in primary neurons.

**Conclusion:**

*Yy*2 expression in the heart and lung is constitutively expressed during embryogenesis and in adult mice. For the first time, developmental changes of *yy2 *transcription became obvious in various areas of the brain. This suggests that yy2 is involved in developmental gene regulation.

## Background

The biology of the transcription factor Yin Yang 2 (YY2) is not well characterized yet, but may be of particular interest due to its similarities to the ubiquitously expressed YY1, which has been implicated in fundamental biological processes such as DNA-replication, cell cycle regulation and organogenesis [1-5]. YY2 is a zinc finger protein that shares 56% sequence homology with YY1 [6]. Especially, the C-terminal DNA-binding zinc finger is highly conserved [6-8] and mediates a similar binding specificity to the 5'-(A/c/g)(A/t)NATG(G/a/t)(C/a)(G/c/t)-3' DNA consensus motif [8,9], suggesting synergistic or competitive functions, if both factors act within the same cell [10]. The biological significance of yy1 is highlighted by early embryonic lethality of mice with homozygous *yy1 *deletion. In addition, mice with heterozygous *yy1 *ablation show growth retardation and defects in neurulation [3].

Analysis of *yy2 *ablation has not been reported, yet. The intron lacking *yy2 *gene is extraordinarily positioned between exons 5 and 6 of another X-chromosomally located gene encoding mbtps2 (membrane bound transcription factor protease site 2). First expression analyses by *in situ *hybridization in testis, ovaries and brain from adult mice suggested a shared control of *yy2 *and *mbtps2 *gene activities [7]. However, our data indicate that the upstream region of the human *YY2 *gene mediates significant promoter activity independently from *MBTPS2 *[11].

To gain more information on the biology of yy2, we established whole mount *in situ *hybridization. In parallel, real-time PCR was used to quantify *yy2 *mRNA expression in various organs of developing and adult mice more precisely. Herein, we demonstrate that the *yy2 *mRNA expression pattern differs from that of *yy1 *and *mbtsp2*. Importantly, *yy2 *expression underlies significant changes during development, particularly in various areas of the brain.

## Results

### Expression of *yy2 *in mouse embryos

**Figure 1:**
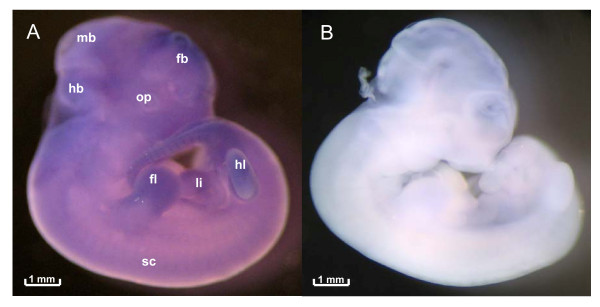
**Expression of *yy2 *in developing mice**. A. Whole mount *in situ *hybridization of E11.5 embryo with a DIG-labeled *yy2 *antisense riboprobe. For a better orientation different regions are indicated; forebrain (fb), midbrain (mb), hindbrain (hb), optical vesicles (op), spinal cord (sc), forelimb buds (fl), liver (li) and hindlimb buds (hl). B. Hybridization with the *yy2 *sense riboprobe served as control (magnification: 4-fold).

Since there are only limited data available on the organ-specific *yy2 *expression [6,7], we established the whole mount *in situ *hybridization technique. First experiments with DIG-labeled riboprobes showed wide spread signals of *yy2 *transcripts on the entire E11.5 embryo (Figure 1A), suggesting ubiquitous expression similar as described for *yy1 *[3]. The specificity of the antisense riboprobe staining is underlined by the negative control, where the embryo was incubated with the corresponding sense riboprobe (Figure 1B). To analyze the *yy2 *expression pattern more precisely, we subsequently performed TaqMan real-time PCR in selected organs and tissue specimens of developing and adult mice.

### Quantitative analysis of *yy2 *expression in heart and lung

**Figure 2:**
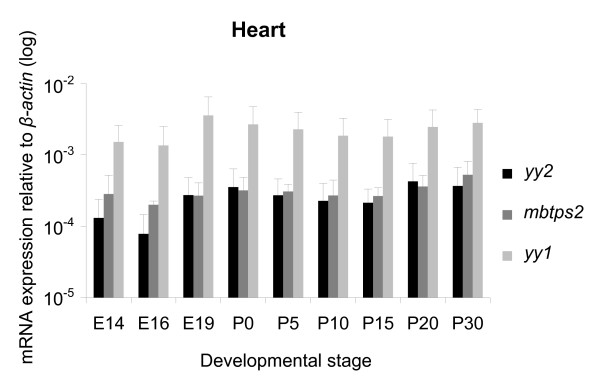
**Quantification of *yy2*, *mbtps2 *and *yy1 *in developing heart**. Expression levels of *yy2*, *mbtps2 *and *yy1 *transcripts in the heart during development and in adult mice (E14 to P30) quantified by TaqMan real-time PCR. The expression is normalized to *β-actin*. All three gene products are constitutively expressed at constant levels throughout all stages. (n = 3 sets).

**Figure 3:**
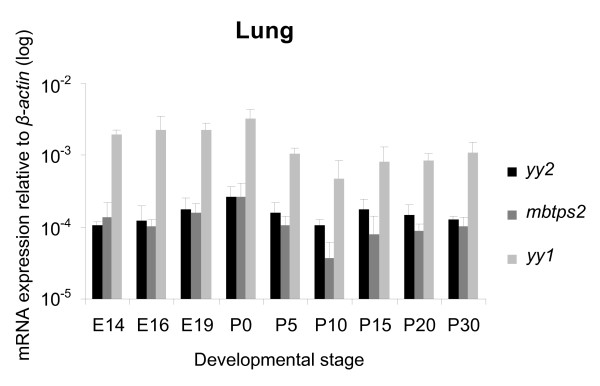
**Quantification of *yy2*, *mbtps2 *and *yy1 *in developing lung**. Quantification of *yy2*, *mbtps2 *and *yy1 *transcripts levels in the lung during embryogenesis and adult stages (E14 to P30) by TaqMan real-time PCR. The expression data are normalized to *β-actin*. The pattern is similar to the heart analyzes. (n = 3 sets).

Specimens of the heart and lung, taken at several developmental stages, were dissected, and cDNAs were amplified out of isolated RNA. We analyzed samples spanning from E14 to P60, since in earlier developmental stage region-specific specimens cannot be isolated due to technical limitations. By using TaqMan real-time PCR technique with FAM-labeled probes, we tested the specimens also for *yy1 *and *mbtps2 *transcripts. For all three transcripts we found almost stable expression level throughout all tested developmental stages of the heart (Figure 2) and lung (Figure 3). However, in contrast to the highly expressed *yy1*, the amount of *yy2 *and *mbtps2 *transcripts was clearly lower in all developmental stages. Interestingly, *yy2 *and *mbtps2 *showed always similar expression levels. Thus, neither the *yy *genes nor *mbtps2 *showed any significant changes in expression during heart and lung development.

### Quantitative analysis of *yy2 *expression in the brain

**Figure 4:**
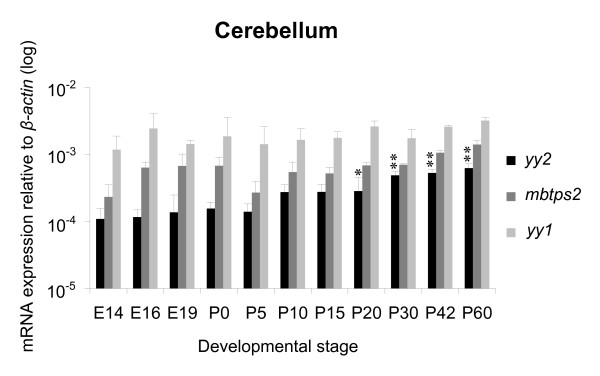
**Quantification of *yy2*, *mbtps2 *and *yy1 *in developing cerebellum**. Analyzes of *yy2*, *mbtps2 *and *yy1 *expression in the cerebellum throughout development (E14 to P60). TaqMan real-time PCR revealed that *mbtps2 *and *yy1 *are constitutively expressed at almost constant levels, whereas *yy2 *expression is low at E14, but increases significantly until P60. Expression levels are normalized to *β-actin*. (n = 3 sets; * *p *< 0.05; ** *p *< 0.01; statistical significances refer to E14).

**Figure 5:**
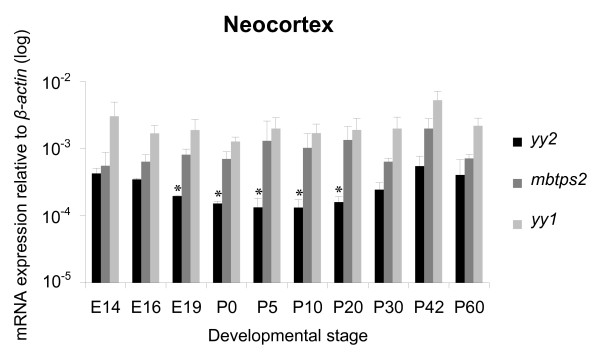
**Quantification of *yy2*, *mbtps2 *and *yy1 *in developing neocortex**. Quantitative expression analyses (TaqMan real-time PCR) of *yy2*, *mbtps2 *and *yy1 *transcripts in the neocortex during development and in adult mice (E14 to P60). The expression of *yy2 *reaches a minimum around day P5. Expression levels are normalized to *β-actin*. (n = 3 sets; * *p *< 0.05; ** *p *< 0.01; statistical significances refer to E14).

**Figure 6:**
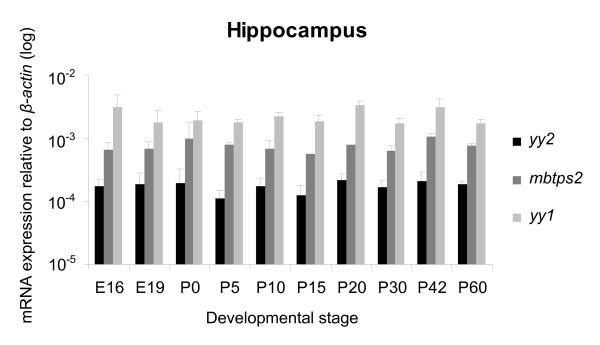
**Quantification of *yy2*, *mbtps2 *and *yy1 *in developing hippocampus**. Expression levels of *yy2*, *mbtps2 *and *yy1 *transcripts in the hippocampus during development and in adult mice (E14 to P30) quantified by TaqMan real-time PCR. The expression is normalized to *β-actin*. All three gene products are constitutively expressed at constant levels throughout all stages. (n = 3 sets).

Previously, the expression of *yy2*, *yy1 *and *mbtps2 *in the neocortex and cerebellum of adult mice has been shown by *in situ *hybridization, but not quantified [7]. To analyze the gene expression during development we used the quantitative real-time PCR technique. As shown in Figure 4 and 5, there were significant changes of *yy2 *expression from day E14 to P60 by means of TaqMan real-time PCR. By contrast, the *yy1 *levels remained stable. Interestingly, in cerebellum the amount of *yy2 *increased up to 6-fold with maturation. In the neocortex *yy2 *expression significantly decreased during intrauterine gestation, reached a minimum in the early neonatal period, before it increased again in adult mice (P60). However, the content of *yy2 *transcripts in the hippocampus remained stable on a lower level compared to those of cerebellum and neocortex (Figure 6). In all brain regions, *mbtps2 *and *yy1 *were consistently higher expressed (up to 10-fold) than *yy2*. In contrast to *yy2*, transcripts of *yy1 *and *mbtps2 *were almost constitutively expressed during development. The combined data indicate that *yy2 *expression underlies a tissue- and developmental-stage specific regulation.

### Quantitative analysis of *yy2 *expression in neurons, astrocytes and microglia

**Figure 7:**
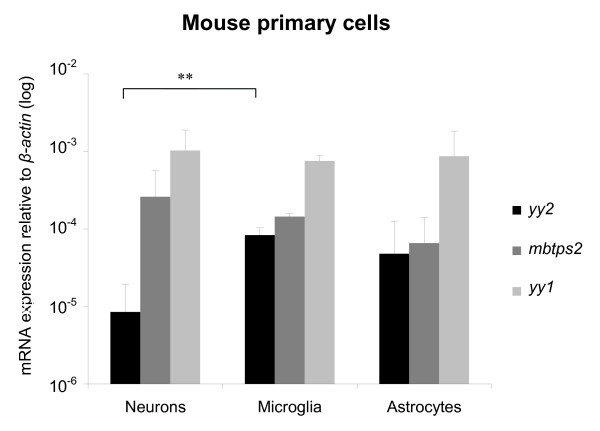
**Quantification of *yy2*, *mbtps2 *and *yy1 *in primary cells**. Expression levels of *yy2*, *mbtps2 *and *yy1 *transcripts in the different primary cells (neurons, microglia and astrocytes) quantified by TaqMan real-time PCR. While *mbtps2 *and *yy1 *are strongly and constantly expressed in all cell types, *yy2 *is significantly reduced in neurons. Expression values are normalized to *β-actin*. (n = 3; ** *p *< 0.01).

Due to the dynamic regulation of *yy2 *expression in neocortex and cerebellum, we analyzed the expression of *yy2*, *yy1 *and *mbtps2 *transcript levels in primary neurons, astrocytes and microglia of mice by using real-time PCR. As depicted in Figure 7, *yy1 *was strongly expressed at similar levels within all three cell types, whereas *yy2 *and *mbtps2 *were found at lower levels. Especially neurons contained a very low amount of *yy2 *transcript, if compared to astrocytes and microglia cells [see also additional file 1].

## Discussion

Herein, for the first time we provide data implying that the transcription factor yy2 possess a functional role during development, as it has already been shown for its famous and well characterized homologue yy1 [3,12,13]. Since, *yy2 *is broadly, possibly ubiquitously expressed in embryonic mice (Figure 1), we conducted extensive real-time PCR analyses indicating a tissue-specific expression pattern that is in part developmentally regulated. Thereby, our experiments were especially focused on the cardiopulmonary and central nervous system.

**Figure 8:**
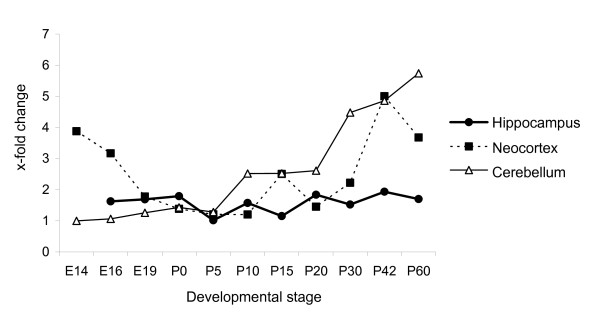
**Comparison of *yy2 *expression in different brain regions**. Comparison of *yy2 *expression levels in the hippocampus, neocortex and cerebellum. E14 from cerebellum (lowest starting value) was defined as one. While expression of *yy2 *is stable in the hippocampus throughout all stages, the amount of *yy2 *transcripts varies significantly in cerebellum and neocortex. (n = 3 sets)

Expression of *yy1*, *yy2 *and *mbtps2 *could be detected in all analyzed organs, tissues and cells [see also additional file 2]. While their expression levels did not change significantly during development of heart and lung, the specimens from the brain revealed a more dynamic expression pattern. The relative high amount of *yy1 *transcripts in the cerebellum, neocortex and hippocampus underlines the broad implication of yy1 in the brain at all stages of mice development [14]. In fact, its functional role in the nervous system has been demonstrated by analyses of heterozygous *yy1 *knock-out mice [3,12] and conditional ablation of *yy1 *in oligodendrocytes [15]. By contrast, the amount of *yy2 *showed tissue-specific variations throughout brain development (summarized in Figure 8). In the cerebellum, *yy2 *expression increased significantly from early postnatal to adult. During this time period cerebellum undergoes dramatic morphological changes, including active neurogenesis, neuronal migration and differentiation [16]. In cortical samples *yy2 *expression revealed a u-shaped progression with a minimum starting at birth. At this time point neurogenesis and neuronal migration are completed in the cortex [17]. Here, it has to be noted that cerebellum and cortex have specific parallel neuronal connections between each other [18]. For example, neurons from the motor cortex project to the cerebellum. The hippocampus, however, involved in learning and memory processes, exhibited a constant low level of *yy2 *transcripts throughout development. Further analyses of yy2 protein localization at the cellular level could help to understand whether yy2 could be involved in controlling axon formation, migration, or dendritogenesis. This issue cannot be examined so far, because currently no appropriate antibody against murine yy2 is available.

By analyzing different primary cell types isolated from the murine brain, we found differences of *yy2 *expression as well, whereas *yy1 *levels showed no significant changes. In contrast to microglia cells and astrocytes, neurons express almost no *yy2*. The neuronal data suggest that yy2 could be required for proliferation, since neurons, in contrast to astrocytes and microglia cells, cannot divide anymore. Ongoing experiments may verify this hypothesis.

Comparing all real-time PCR results it becomes obvious that the expression of *yy2 *and *mbtps2*, respectively, is individually regulated depending upon the analyzed organ, tissue or cell type. While both genes seem to be co-regulated *via *the same promoter in heart and lung as well as in microglia and astrocytes, however, the data from primary neurons and all three brain tissues indicate an alternative transcriptional activity. Currently, two different possibilities of *yy2 *and *mbtps2 *gene regulation are discussed: (a) both genes are under the control of the *mbtps2 *promoter, and (b) *yy2 *transcription is mediated by its own adjacent promoter, located upstream of its coding sequence, a model that we favour according to own data on the regulation of the human *YY2 *gene [7,11]. Of note, only the *yy2 *promoter shows a significant sensitivity against DNA-(de)methylation, strengthening the implication of yy2 in developmental processes. Our observations presented in this manuscript emphasize that in fact both promoter regions seem to be utilized in a tissue- or cell type-specific manner.

## Conclusion

The results from the real-time PCR experiments confirm that *yy2 *is differentially expressed in the lung, heart and brain, indicating a stringent spatial regulation and function. Precisely, we show a significant change of *yy2 *expression during the development and maturation of the neocortex and cerebellum. Within the central nervous system, *yy2 *is predominantly expressed in astrocytes and microglia cells.

## Methods

### Animals

C57/Bl6 mice were mated, and embryos/pups were collected at defined time points (morning of vaginal plug was considered as embryonic day E0.5). The protocol for animal use was approved by the Institutional Review Board (T 0167/08).

### Preparation of primary neurons

Serum-free preparation of mouse cortical primary cultures was performed with E18 (+/- 0.5 days) mouse embryos as previously described [19]. After removal of meninges, entire cortices were mechanically dissociated in HBSS buffer (w/o Ca^2^^+ ^and Mg^2^^+^), with trypsin (0.25%) for 10 min at 37°C. Trypsinization was stopped by adding minimum essential medium (Gibco-Invitrogen; Karlsruhe, Germany). Medium was supplemented with 0.6% glucose, 10% horse serum (Gibco), and penicillin and streptomycin. Further mechanical dissociation was performed by adding of DNAseI (0.06%). Cells were cultured on poly-L-lysine-coated dishes at a density of 75.000 cells/cm^2^. After three hours *in vitro*, medium was changed to Neurobasal A medium (Gibco) supplemented with 2% B27 (Gibco), 0.5 mM glutamine, and penicillin and streptomycin for 12 days. Purity of neuronal cell preparation was tested by real-time PCR [see additional file 3].

### Preparation and purification of astrocytes and microglial cells

For astrocyte and microglia preparation, cerebra from mouse pups aged P1 to P3 were prepared and the meninges, including the pia mater and arachnoid, were carefully removed and the cerebrum was washed in DMEM (4.5 g/l glucose, 200 mM glutamine, pyrovate) containing 10% fetal calf serum and 100 U/ml penicillin/streptomycin. Following careful homogenization with a fire-polished Pasteur pipette, a 10 min trypsin/DNAse (1.25%/2 U) incubation followed, and digestion was stopped by adding the same volume culture medium (DMEM containing 4.5 g/l glucose, 200 mM glutamine, pyrovate, 10% fetal calf serum, and 100 U/ml penicillin/streptomycin). Dissociated astrocytes were plated on poly-L-lysine (10 μg/ml)-coated dishes. Every third day, dishes were shaken for 10 min at 37°C and afterwards rinsed three times with warm PBS to remove microglial cells. They were then resuspended with a fire-polished Pasteur pipette, and replated in culture medium at low density. The suspended microglial cells were collected and replated in astroglial culture medium containing 2% fetal calf serum. Purity of both cell types was tested by real-time PCR [see additional file 3].

### Whole-mount *in situ *expression analysis of *yy2 *mRNA

For *in situ *hybridization, a *yy2 *probe was generated by cloning a *yy2*-specific 200 bp fragment (nt 11 to 200) derived from the murine coding sequence [GenBank: EF688658] into pBluescript SK (-) plasmid (Fermentas; St. Leon-Rot, Germany). For digoxygenin (DIG)-labeling, 12 μl of linearized plasmid (EcoRI: anti-sense or BamHI: sense) were combined with 2 μl 10-fold buffer (Roche; Mannheim, Germany), 1 μl RNAsin, 2 μl T3 (for anti-sense synthesis) or T7 (for sense synthesis) RNA polymerase and 2 μl labeling mix containing 7.1 μl ATP, 7.1 μl CTP, 7.1 μl GTP, 4.6 μl UTP, 20.5 μl RNase free water, and 25 μl DIG-11-UTP (Roche; Cat.-No.: 1209256) in order to conduct *in vitro *transcription for 2 h (37°C). Afterwards, the freshly prepared probe was diluted by adding 70 μl RNase free water and 10 μl 0.1 M DTT. The embryos were dissected in ice-cold PBS, followed by overnight fixation in 4% PFA, 3 washes in PBS with 1% Tween 20 (PBT) for 5 minutes, and dehydration in rising methanol concentrations. Next, specimens were rehydrated with decreasing methanol concentrations. For increasing permeability and signal detection, embryos were incubated in 6% H_2_O_2 _for 1 h and subsequently treated with proteinase K for 15 min with 3 washing steps (5 min each) in PBT. Digestion was stopped in 2 mg/ml glycine in PBT for 20 min and 2 washing steps in PBT followed by post fixation in 4% PFA (in PBT) for 20 min at room temperature. PFA was removed by 3 washing steps in PBT. After incubation in hybridization solution containing 50% deionized formamide, 5× SSC [pH 4.5], 1% SDS, 50 μg/ml tRNA and 50 μg/ml heparin at 65°C for 1 h, 1–2 μl denaturized DIG-labeled probes (2 min at 90°C) were added and hybridized at 65°C overnight. Finally, embryos were washed 3 times for 1 h in solution I (50% formamide, 5 × SSC [pH 4.5], 1% SDS) at 65°C followed by 3 washing steps for 1 hour in solution II (50% formamide, 2 × SSC [pH 4.5], 1% Tween 20) at 60°C and washing 3 times in TBST for 5 min each. Before the embryos were treated with anti-DIG antibody (1:2000) in 1% sheep serum/TBST overnight at 4°C, we conducted a blocking step in 10% heat-inactivated sheep serum/TBST with 0.1% blocking reagent (Roche; Cat.-No.: 11112589001) for 1 h at room temperature. After performing post-antibody washes (3 times 10 min and 5 times 1 h in TBST), samples were hybridized in fresh prepared NTMT [pH 9.5] solution containing 100 mM NaCl, 100 mM Tris-Cl (pH 9.5), 50 mM MgCl_2 _and 0.1% Tween 20. For signal detection, NTMT solution was removed and reaction mix (NTMT with 125 μg/ml BCIP and 250 μg/ml NBT) was added. To stop the staining process, samples were washed 3 times in PBT, followed by post-fixation in 4% PFA. The embryos were analyzed with a Leica MZ 9.5 stereo microscope (Wetzlar, Germany).

### Preparation of cDNA

Mouse organs were immediately snap-frozen following their collection from 3 sets of 6 animals of each age. Primary mouse brain cells from 3 independent preparations of 3 pregnant animals or mouse organs were homogenized in TRIzol reagent (Invitrogen), and the total RNA was purified according the TRIzol protocol. RNA concentrations were determined by using a UV-Visible Spectrophotometer (Biomate 3 spectrometer, Fisher Scientific, Waltham, MA, USA). A High Capacity cDNA Reverse Transcription kit (Applied Biosystems, Foster City, CA, USA) was used to generate total cDNA for the real-time PCR from 5 μg total RNA from each sample as per the manufacturer's recommendations.

### Real-time PCR (TaqMan)

TaqMan cDNA samples were prepared as described. 5'-FAM-labeled probes and appropriate primer pairs for detection of murine *yy2 *(probe: 6FAM-cagcctgttcttcagctatgggatcttctt-BBQ; primer: 5'-gggtgacaaacagtgggagc-3' (forward) and 5'-ggatcagaaagatcaatgccaggt-3' (reverse)), *yy1 *(probe: 6FAM-agggtctgagaggtcaatgccaggt-BBQ; primer: 5'-atgaaacagtggttgaagagcagatc-3' (forward) and 5'-caagctattgttcttggagcatcatc-3'(reverse)) and *mbtps2 *(probe: 6FAM-tgtcccgttactaatgtgcaagattggaa-BBQ; primer: 5'-ggagaccttgtcactcatctacagga-3' (forward) and 5'-gtcgtttgtatgctctaactgggaag-3' (reverse)) were designed and synthesized by TIB MOLBIOL (Berlin, Germany). *β-actin *(TaqMan Gene Expression Assays; Cat.-No.: ACTB 4352664-0602004) was purchased from Applied Biosystems. For all PCR reactions, 1 μl of cDNA was added to 10 μl TaqMan 2× Universal PCR Mastermix Mix (Applied Biosystems), 1 μl of each primer [20 μM], 1 μl 5'-FAM-labeled probe [0.3 μM] and 8 μl deionized H_2_O. All reactions were performed in duplicates and all three transcripts (within each set of specimens) were always analyzed within the same experiment. Amplification and fluorescence detection was conducted with the i-Cycler Multicolor Real-Time PCR Detection System (Bio-Rad; Munich, Germany). The fluorescence threshold value was calculated by using the iCycle iQ Optical System Software, version 3.1. *yy2, yy1 and mbtps2 *values were normalized against *β-actin*.

### Statistical analysis

Statistical significances were determined by using the one-way analysis of variance (ANOVA). Bonferroni's multiple comparison procedure was used to discriminate, which means were different from others. A *p*-value < 0.05 was considered to be significant.

## Authors' contributions

DD established, performed and analyzed *in situ *hybridizations, performed real-time PCR, drafted and edited the manuscript. MK and CD designed the project, supervised DD, contributed to the analysis of *in situ *hybridization as well as real-time PCR and edited the manuscript. AUB provided cDNA samples, contributed to analysis of *in situ *hybridization and real-time PCR. All authors read and approved the final version of the manuscript.

## Supplementary Material

Additional file 1 **Detection of *yy2*, *mbtps2 *and *yy1 *in primary cells**. Expression of *yy2*, *mbtps2 *and *yy1 *in the primary cells (neurons, microglia and astrocytes) isolated from the murine brain were analyzed by conventional PCR (30 cycles per reaction; *yy2*: 5'-accagcgtaggccaaaccatcgaagta-3' (forward) and 5'-cgtcaaaccacagagattcccttcata-3' (reverse); *β-actin*: 5'-actgctctggctcctagcac-3' (forward) and 5'-acatctgctggaaggtggac-3' (reverse); *mbtps2*: 5'-ggagaccttgtcactcatctacagga-3' (forward) and 5'-gtcgtttgtatgctctaactgggaag-3' (reverse); *yy1*: 5'-atgaaacagtggttgaagagcagatc-3' (forward) and 5'-caagctattgttcttggagcatcatc-3'(reverse)). While *mbtps2 *and *yy1 *are strongly and constantly expressed in all cell types, *yy2 *is reduced in neurons. Expression of *β-actin *served as internal control (multiplex PCR; upper panel). *n = 3* Click here for file

Additional file 2 **Expression of *yy2*, *mbtps2 *and *yy1 *in testis, liver, kidney, thymus and spleen**. Levels of *yy2*, *mbtps2 *and *yy1 *in the different stages of testis, liver, kidney and spleen development (E16, P10 and P30) were analyzed by conventional PCR (30 cycles per reaction;*yy2*: 5'-accagcgtaggccaaaccatcgaagta-3' (forward) and 5'-cgtcaaaccacagagattcccttcata-3' (reverse); *β-actin*: 5'-actgctctggctcctagcac-3' (forward) and 5'-acatctgctggaaggtggac-3' (reverse); *mbtps2*: 5'-ggagaccttgtcactcatctacagga-3' (forward) and 5'-gtcgtttgtatgctctaactgggaag-3' (reverse); *yy1*: 5'-atgaaacagtggttgaagagcagatc-3' (forward) and 5'-caagctattgttcttggagcatcatc-3'(reverse)). With the exception of thymus for *yy2 *and testis regarding *mbtps2*, both gene products are differentially regulated in all tested organs. However, *yy1 *expression only showed developmental changes in liver and spleen. Expression of *β-actin *served as internal control (multiplex PCR; upper panel). *n = 3* Click here for file

Additional file 3 **Purity of primary cells**. The Table shows the means of relative expression including standard deviation (SD) of specific marker genes as *β3-tubulin *(*Tuj-1*) for neurons, *ionized calcium-binding adapter molecule-1 *(*Iba-1*) for microglia and *glial fibrillary acidic protein *(*GFAP*) for astrocytes related to *β-actin *(probes: *Tuj-1 *Cat.-No.: Mm00727586_s1; *Iba-1 *Cat.-No.: Mm00479862_g1; *GFAP *Cat.-No.: Mm00546086_m1; *β-actin*: Cat.-No.: 4352933E; Applied Biosystems) determined by real-time PCR. Click here for file
