# Supplementary figures and images for: Developmental expression profile of the yy2 gene in mice
Source: BMC Dev Biol. 2009 Jul 28;9:45. doi: 10.1186/1471-213X-9-45 (PMC2724487; doi:10.1186/1471-213X-9-45)

Ladder

Neurons

Microglia

Astrocytes

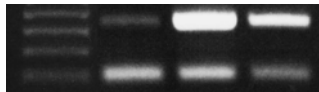

← *yy2*

←  $\beta$ -actin

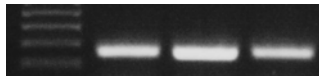

← *mbtps2*

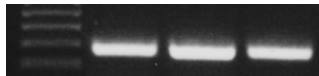

← *yy1*

Supplement: Additional file 1 — Detection of yy2, mbtps2 and yy1 in primary cells. Expression of yy2, mbtps2 and yy1 in the primary cells (neurons, microglia and astrocytes) isolated from the murine brain were analyzed by conventional PCR (30 cycles per reaction; yy2: 5'-accagcgtaggccaaaccatcgaagta-3' (forward) and 5'-cgtcaaaccacagagattcccttcata-3' (reverse); β-actin: 5'-actgctctggctcctagcac-3' (forward) and 5'-acatctgctggaaggtggac-3' (reverse); mbtps2: 5'-ggagaccttgtcactcatctacagga-3' (forward) and 5'-gtcgtttgtatgctctaactgggaag-3' (reverse); yy1: 5'-atgaaacagtggttgaagagcagatc-3' (forward) and 5'-caagctattgttcttggagcatcatc-3'(reverse)). While mbtps2 and yy1 are strongly and constantly expressed in all cell types, yy2 is reduced in neurons. Expression of β-actin served as internal control (multiplex PCR; upper panel). n = 3 [file 1471-213X-9-45-S1.pdf]

Testis

Liver

Kidney

Thymus

Spleen

Ladder

E16

P10

P30

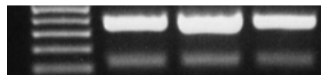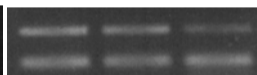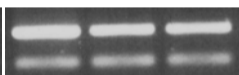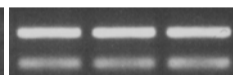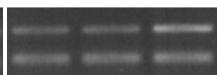

← *yy2*  
← *β-actin*

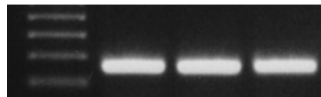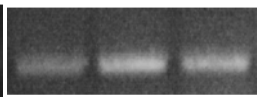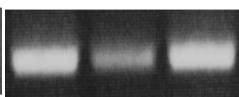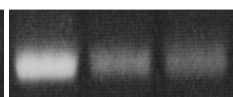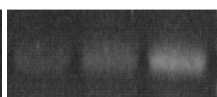

← *mbtps2*

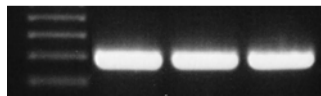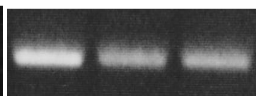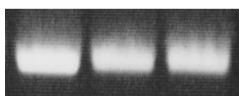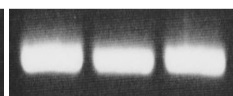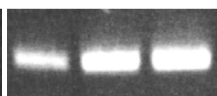

← *yy1*

Supplement: Additional file 2 — Expression of yy2, mbtps2 and yy1 in testis, liver, kidney, thymus and spleen. Levels of yy2, mbtps2 and yy1 in the different stages of testis, liver, kidney and spleen development (E16, P10 and P30) were analyzed by conventional PCR (30 cycles per reaction;yy2: 5'-accagcgtaggccaaaccatcgaagta-3' (forward) and 5'-cgtcaaaccacagagattcccttcata-3' (reverse); β-actin: 5'-actgctctggctcctagcac-3' (forward) and 5'-acatctgctggaaggtggac-3' (reverse); mbtps2: 5'-ggagaccttgtcactcatctacagga-3' (forward) and 5'-gtcgtttgtatgctctaactgggaag-3' (reverse); yy1: 5'-atgaaacagtggttgaagagcagatc-3' (forward) and 5'-caagctattgttcttggagcatcatc-3'(reverse)). With the exception of thymus for yy2 and testis regarding mbtps2, both gene products are differentially regulated in all tested organs. However, yy1 expression only showed developmental changes in liver and spleen. Expression of β-actin served as internal control (multiplex PCR; upper panel). n = 3 [file 1471-213X-9-45-S2.pdf]
