# Supplementary material for: Developmental expression profile of the yy2 gene in mice
Source: BMC Dev Biol. 2009 Jul 28;9:45. doi: 10.1186/1471-213X-9-45 (PMC2724487; doi:10.1186/1471-213X-9-45)
Supplement: Additional file 3 — Purity of primary cells. The Table shows the means of relative expression including standard deviation (SD) of specific marker genes as β3-tubulin (Tuj-1) for neurons, ionized calcium-binding adapter molecule-1 (Iba-1) for microglia and glial fibrillary acidic protein (GFAP) for astrocytes related to β-actin (probes: Tuj-1 Cat.-No.: Mm00727586_s1; Iba-1 Cat.-No.: Mm00479862_g1; GFAP Cat.-No.: Mm00546086_m1; β-actin: Cat.-No.: 4352933E; Applied Biosystems) determined by real-time PCR. [file 1471-213X-9-45-S3.pdf]

	Tuj-1 ± SD	*Iba-1* ± SD	*GFAP* ± SD
Neurons	0,24728 ± 0,03513	0,00002 ± 0,00001	0,03323 ± 0,00548
Microglia	0,00072 ± 0,00025	0,04826 ± 0,01443	0,03788 ± 0,04975
Astrocytes	0,00101 ± 0,00061	0,00549 ± 0,00411	0,90779 ± 0,19163
